# Systemic administration of 3-bromopyruvate reveals its interaction with serum proteins in a rat model

**DOI:** 10.1186/1756-0500-6-277

**Published:** 2013-07-17

**Authors:** Rani Kunjithapatham, Jean-Francois H Geschwind, Pramod P Rao, Tatiana N Boronina, Robert N Cole, Shanmugasundaram Ganapathy-Kanniappan

**Affiliations:** 1 Russell H. Morgan Department of Radiology and Radiological Sciences, Johns Hopkins University School of Medicine, 600 N. Wolfe Street, Baltimore, MD 21287, USA; 2 Mass Spectrometry and Proteomics Facility, Johns Hopkins University School of Medicine, 733, N. Broadway Street, Baltimore, MD 21205, USA

**Keywords:** 3-bromopyruvate, LC-MS/MS, Alpha1 antitrypsin, 2D gel electrophoresis

## Abstract

**Background:**

3-bromopyruvate (3-BrPA) is a glycolytic inhibitor that affects cancer cells by targeting energy metabolism. Preclinical reports have established that a 1.75 mM dose of 3-BrPA is effective and sufficient to inhibit tumor growth when administered under a loco-regional approach (intraarterial and intratumoral). This loco-regional therapeutic dose was found to be nontoxic when given systemically as well. Yet, the mechanism underlying this lack of toxicity of 1.75 mM 3-BrPA during systemic delivery is unknown. Here, we investigated the mechanism associated with the lack of organ toxicity when 1.75 mM 3-BrPA was administered systemically using radiolabeled (^14^C)-3-BrPA in Sprague–Dawley rats.

**Findings:**

Data obtained from tissue-autoradiography of rats infused with ^14^C-3-BrPA showed strong ^14^C-signal in tissue sections of various organs except the brain corroborating that 3-BrPA does not cross the blood–brain barrier. Significantly, Hematoxylin & Eosin staining and apoptosis assay of tissue sections positive for ^14^C-signal showed no signs of toxicity or apoptosis. Convincingly, the ^14^C-signal observed in tissue-autoradiography emanates from 3-BrPA that is non-reactive or non-toxic, hence we further investigated whether the lack of toxicity is due to its interaction or alkylation with serum components. Analysis of serum proteins by 1D and 2D-gel electrophoretic autoradiography showed that ^14^C-BrPA selectively binds to peptides of molecular mass ~50-60 kDa. Mass spectrometry data suggested that ^14^C-BrPA could interact with alpha1-antitrypsin and a peptide of albuminoid-family.

**Conclusion:**

Our data indicate that selective interaction of 3-BrPA with serum proteins could contribute to the apparent lack of tissue-toxicity at the indicated close when the drug is given systematically in Sprague–Dawley rats.

## Findings

### Background

Recent reports have demonstrated the therapeutic potential of targeting energy metabolism in cancer cells [1-3]. Consequently, research on potent inhibitors of (aerobic) glycolysis, a major energy producing pathway, has gained renewed interest [4]. The pyruvate analog, 3-bromopyruvate (3-BrPA) is a glycolytic inhibitor that affects cancer cells by disrupting energy metabolism [5]. Studies from our laboratory and others have identified the principal intracellular targets and molecular mechanisms involved in 3-BrPA’s antitumor effects [6-8]. 3-BrPA irreversibly alkylates the glycolytic enzyme, glyceraldehyde-3-phosphate dehydrogenase (GAPDH) resulting in the disruption of glucose metabolism leading to cell death. Several studies have demonstrated the therapeutic advantage of 3-BrPA against different types of cancers, *in vitro* and *in vivo*[9-15]. Further investigations on animal tumor models demonstrated that 1.75 mM 3-BrPA is the effective therapeutic dose for treating liver cancer through loco-regional approaches such as intra-arterial (IA) or intratumoral deliveries [14,16]. Thus, substantial preclinical data and a wealth of information on the molecular mechanisms of 3-BrPA have highlighted its potential as an effective agent for cancer treatment.

Several antineoplastic alkylating agents (e.g. cisplatin, oxaliplatin) have been known to interact with serum proteins upon systemic administration [17,18]. However, there is paucity of data indicating such interactions of 3-BrPA with any of the serum proteins. Although 3-BrPA differs considerably from the majority of alkylating agents in its mode of alkylation and its anticancer mechanism, any insight on the interaction of 3-BrPA with serum proteins would greatly improve our ability to use 3-BrPA systemically.

Previously we have shown that in the rabbit Vx-2 liver tumor model, an IA therapeutic dose (1.75 mM concentration) of 3-BrPA did not affect normal liver parenchyma surrounding the tumor [16]. Notably, systemic administration of the IA therapeutic dose of 3-BrPA to tumor-bearing rabbits did not show any sign of toxicity [19]. Yet, the mechanism underlying the lack of systemic toxicity of 3-BrPA during systemic delivery remains unknown. In this report, we investigated the possible mechanism(s) associated with the lack of organ toxicity of systemically administered 3-BrPA using radiolabeled (^14^C)-3-BrPA in Sprague–Dawley rats.

## Results and discussion

Data from the present study demonstrate that the glycolytic inhibitor, 3-BrPA selectively interacts with serum proteins. Further, it is also evident that such an interaction between 3-BrPA and serum proteins could explain the lack of toxicities when the drug is given systemically at least at the 1.75 mM dose.

### Systemic administration of IA therapeutic dose of 3-BrPA shows no organ toxicity

**Figure 1:**
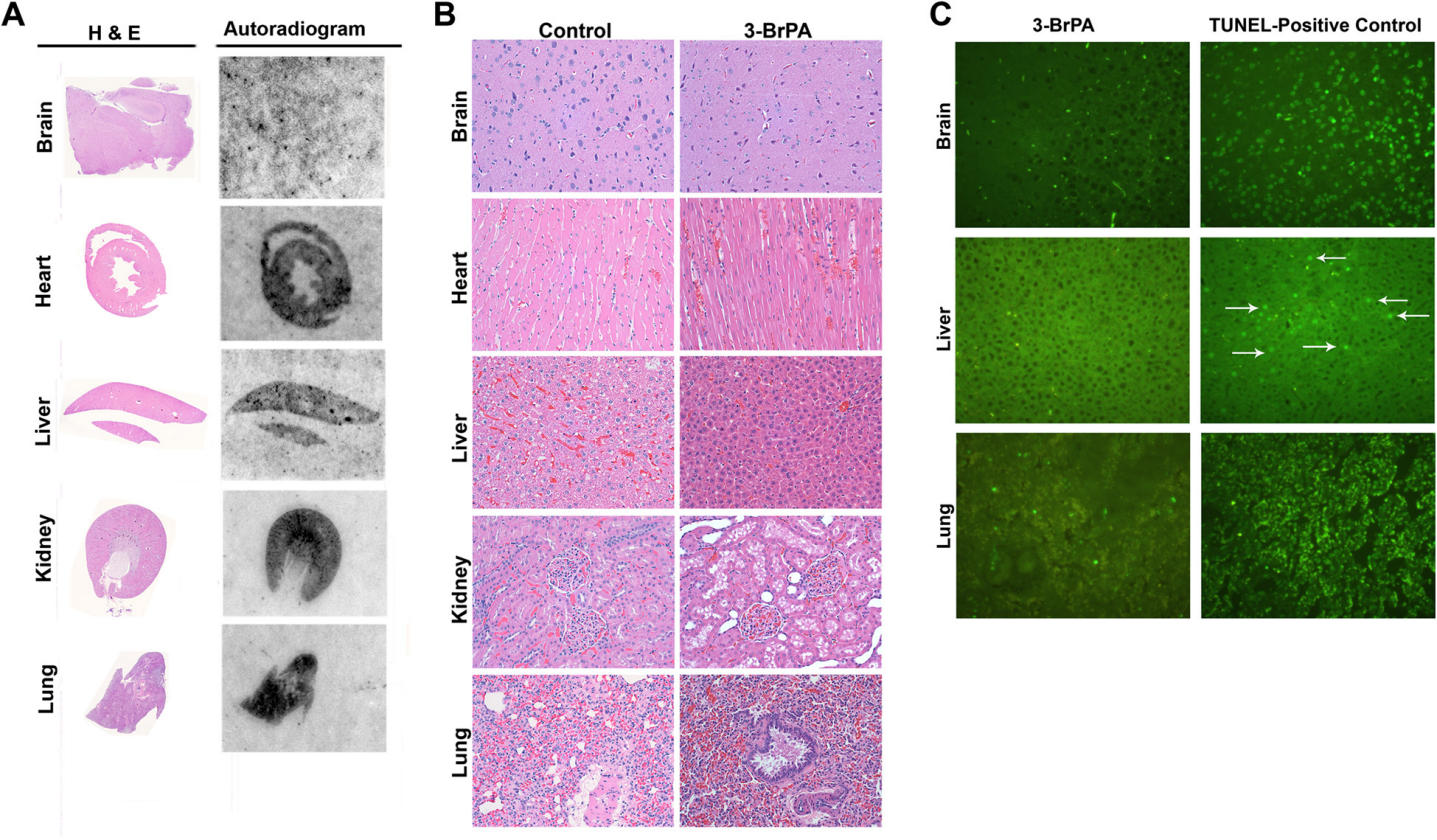
**Distribution of **^**14**^**C-3-BrPA during systemic delivery.** (**A**) Autoradiogram and corresponding H & E staining of tissue sections showing radioactive signal in organs such as liver, lung, kidney and heart but not brain. (**B**) H & E staining showing normal tissue architecture in control and 3-BrPA dosed rat tissues. (**C**) TUNEL-assay showing absence of positive staining in 3-BrPA dosed rat tissues indicating absence of apoptosis. For comparison, TUNEL positive control slides have been included.

Tissue-autoradiography of rats subjected to systemic administration of ^14^C-3-BrPA showed strong radioactive ^14^C-signal in the tissue sections of organs such as heart, liver, kidney and lung, but not the brain (Figure 1A). Evidently, ^14^C-3-BrPA did not cross the blood–brain barrier, which in turn indicates that the neuronal cells could be protected from any toxicity. Remarkably, histopathological analysis of tissue sections that were positive for ^14^C-signal showed normal tissue architecture indicating no signs of toxicity or pathology as evident from H&E staining (Figure 1B). Further, TUNEL staining of the respective tissues from 3-BrPA treated rats showed no positive-staining (Figure 1C), confirming the absence of any apoptosis. Since the tissues were not perfused it is likely that the ^14^C signal observed in tissue sections were from the serum which contained ^14^C-3-BrPA. This is further supported by the gel-electrophoretic autoradiogram where serum proteins showed ^14^C signal. Thus, the ^14^C-signal observed in tissue-autoradiography emanates from 3-BrPA that is non-toxic or non-reactive as it was neutralized or quenched by the interaction with serum proteins.

### Selective binding of ^14^C-3-BrPA with serum proteins

**Figure 2:**
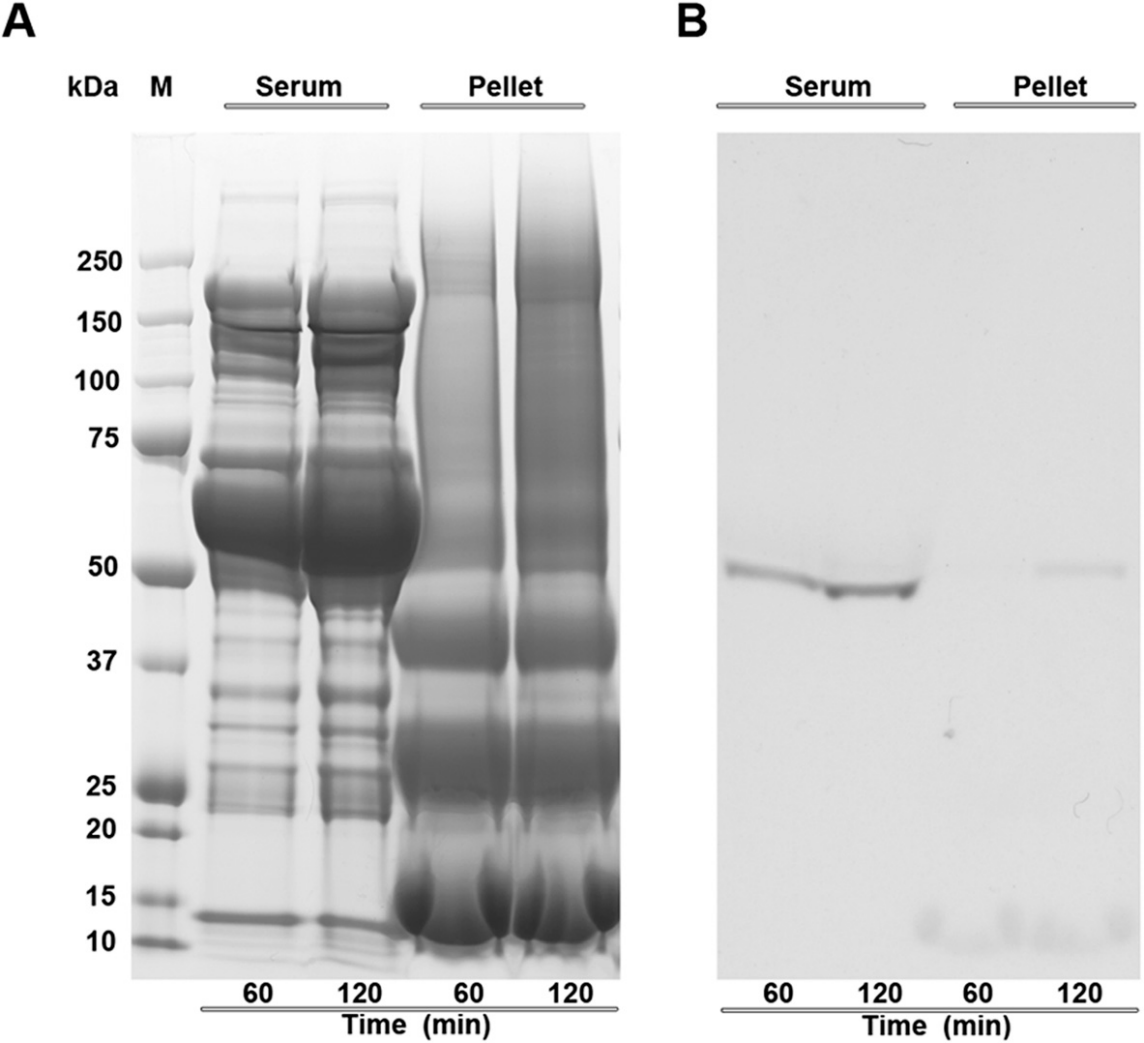
**Selective binding of **^**14**^**C-3-BrPA with rat serum proteins.** (**A**) Coomassie stained SDS-PAGE gel showing the protein profile of serum and pellet of ^14^C-3-BrPA dosed rat. (**B**) A corresponding autoradiogram showing time-dependent increase in autoradiogram signal in the serum, at ~50-60 kDa. Serum obtained from blood collected 60 min or 120 minutes after the 3-BrPA infusion indicate a time dependent increase in the amount of ^14^C incorporation.

**Figure 3:**
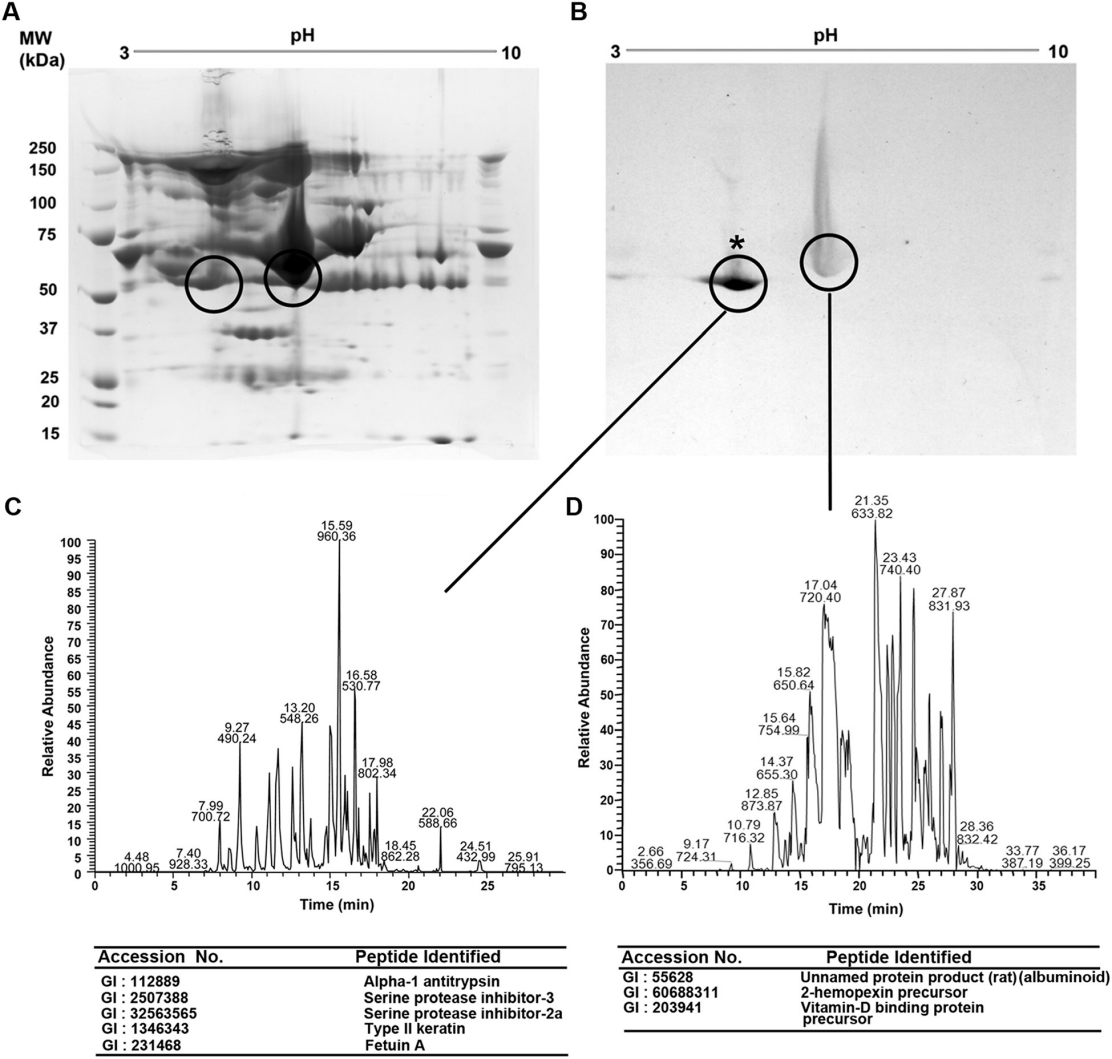
^**14**^**C-3-BrPA primarily binds with two peptides in rat serum.** (**A**) A Coomassie stained 2D-gel showing serum protein spots after 120 minutes of 3-BrPA treatment and (**B**) a corresponding autoradiogram showing a strong and also a weak signal at ~50-60 kDa. (**C**) and (**D**) LC-MS/MS base-peak chromatograms of the peptide spots corresponding to strong (*) and weak signals on 2D-autoradiogram with the list of possible peptides, in the order of matched score.

Autoradiogram of rat serum samples resolved on SDS-PAGE gel demonstrated that systemic administration of ^14^C-3-BrPA resulted in the selective incorporation of ^14^C in rat serum proteins (Figure 2A, B). Interestingly, the pellet (containing erythrocytes and other particulates) did not show any ^14^C incorporation even after 120 minutes of 3-BrPA administration. Based on the ^14^C signal, the 3-BrPA binding has been found to be with the serum peptides of molecular range ~50-60 kDa. Similarly, the autoradiogram of ^14^C-3-BrPA treated rat serum sample resolved on 2D-gel electrophoresis showed significant incorporation of ^14^C selectively in two peptide spots, with strong and weak signals (Figure 3A, B). The autoradiogram signal of the peptide spots from the 2D-gel also localized to the molecular range between ~50-60 kDa, as observed on the one-dimensional SDS-PAGE autoradiogram. Mass Spectrometry identification of the peptide spots corresponding to the strong and weak signals were found to be peptides of alpha-1 antitrypsin (α1-AT) and an albuminoid-family, respectively (Figure 3C, D).

In another experiment, the UV–Vis spectral analysis (180 nm to 800 nm) of mouse serum samples incubated with 3-BrPA *ex vivo*, demonstrated a dose-dependent increase in the absorption maxima of certain serum components at 412, 538 and 572 nm, but not at ~200-204 nm (the absorption maxima of native 3-BrPA) (Additional file 1: Figure S2). The spectral data refer to the total serum components that may include proteins, non-proteinaceous components, small molecules such as glutathione, cysteine, NAD/NADH etc. The objective of the spectral analysis was to demonstrate that 3-BrPA treatment-dependent changes were prominent for the serum components, which was depicted by the pronounced changes at wavelengths such as 412, 538 and 572 nm. The wavelength spectra between 200–204 nm showed the peak absorbance of aqueous 3-BrPA solution that was used as the control. In the serum samples, we did not see any peak at 200–204 nm, which implied that there was no free-3-BrPA present in the serum. Further investigations showed that free-3-BrPA *in vivo* was not detectable by HPLC/mass spectrophotometer even after dose escalation (not shown). The absence of free-3-BrPA *in vivo* as early as 2–3 minutes after systemic administration also provided proof for the immediate reactivity or neutralization of 3-BrPA in serum.

Taken together, the data obtained from proteomic and spectral analyses validate the interaction of 3-BrPA with serum proteins. Although the interaction of anticancer agents such as metallo-drugs with albumin has already been demonstrated [17,18], binding of such agents to α1-AT has not yet been reported, especially with any anti-glycolytic agents. This report is the first to indicate a possible interaction between an anticancer (alkylating) agent (3-BrPA) and α1-AT. α1-AT has been known to be an inhibitor of neutrophil elastase, and this inhibition is required to prevent the enzymatic-degradation of elastin (in lungs). Hence, further studies are required to characterize the impact of 3-BrPA-binding on the inhibitory function of α1-AT.

### Conclusion

Given the promising pre-clinical results on the therapeutic efficacy and mechanism(s) of action of 3-BrPA, the potential exists for translation into the clinic. As a result, it is imperative to understand the possible toxic side effect of 3-BrPA, especially if systemic administration is being contemplated. Our previous report showed that in the rabbit Vx-2 tumor model a dose that was effective given IA did not cause any significant systemic toxicity [19]. As our findings demonstrate the interaction of 3-BrPA with serum proteins, it is likely that the particular interacting 3-BrPA molecule will no longer be available for further alkylation or toxicity. Further, owing to the irreversible alkylating property of 3-BrPA, it is unlikely that the 3-BrPA might be released from these proteins at later stages to contribute any toxicity.

Thus, this report provides an explanation for the apparent lack of systemic toxicity, which could prove extremely useful when considering the optimization of systemic therapy with 3-BrPA.

## Methods

### *In vivo* (systemic) infusion of 3-BrPA to rats

All animals were housed at The Johns Hopkins University Animal Facility, and handled according to the guidelines of NIH and protocols approved by the Institutional Animal Care and Use Committee. Male Sprague Dawley rats (400 to 500 grams body weight) were purchased from Charles River Laboratory (USA) and maintained on automated 12-hour dark/light cycles and allowed access to food and water *ad libitum*.

The radio-(^14^C)-labeled 3-BrPA was synthesized as described previously [19]. Rats were subjected to catheterization procedure (as described in “Additional file 1”) in order to infuse (^14^C)-3-BrPA and to draw blood at different time intervals. Three rats were used for this preliminary study. The experiments were initiated by the systemic administration of a single dose of (^14^C)-3-BrPA (700 μl of 1.75 mM solution) via the femoral vein (Additional file 1: Figure S1). The volume of the dose was determined based on the principle that the administered drug-volume not to exceed 3.5% (i.e. the median of 2-5%) of the total rat-blood volume. Blood samples were collected via the carotid artery at pre-treatment and different time points after (^14^C)-3-BrPA infusion. Blood samples were collected in 1.5 mL eppendorf tubes, allowed to stand at room temperature for 20–30 minutes to form serum separation, followed by centrifugation at 1200 rpm for 20 minutes at 4°C. The clear serum-supernatants were stored at or below −20°C until further analysis. The tissues were harvested at the end of blood collection (120 minutes) by sacrificing the experimental animal, and used for histopathology and autoradiographic analysis.

### Histopathology and tissue autoradiography

For histology, tissues from rat organs such as heart, lung, liver, kidney and brain were routinely fixed in phosphate-buffered 10% formalin (Polysciences Co., Warrington, PA), dehydrated by graded ethanol, embedded in Paraplast Plus wax (McCormick Scientific), sectioned at 5 microns, mounted on slides and oven dried and deparaffinized. The tissue sections were subjected to Hematoxylin and Eosin (H&E) staining as described earlier [14] and viewed under a light microscope. Apoptosis was investigated using terminal deoxynucleotidyl transferase dUTP nick-end labeling (TUNEL) assay kit (Millipore Corp., Bedford, MA) following the manufacturer’s protocol. Finally, tissue sections were covered with cover slips and mounted with Prolong Gold Antifade Reagent (Invitrogen) and allowed to dry in the dark. The slides were viewed under fluorescent microscopy and the images were captured using Nikon Coolpix digital camera (Nikon Instruments, Inc., Melville, NY). Tissue sections from each rat were used for histopathology analysis.

For tissue autoradiography, the unstained histology slides derived from ^14^C-3-BrPA infused rat organs were placed in a large x-ray cassette with intensifying screens, and exposed to x-ray film for 30 days at −80°C followed by film developing.

### SDS-PAGE, 2D gel electrophoresis and autoradiography

The SDS-PAGE as well as 2D gel electrophoresis was performed as described earlier [6]. In brief, serum protein concentration was determined using a 2D-Quant kit (GE- Healthcare, Piscataway, NJ). One-dimensional electrophoresis (SDS-PAGE) was performed using NuPAGE Bis-Tris 4-12% gels followed by colloidal Coomassie blue staining [20]. The samples for 2D gel electrophoresis were cleaned-up using a 2D-Clean-up kit (GE-Healthcare). Isoelectric focusing was performed using Immobiline™ dry gel strips of linear pI (isoelectric point) range 3–10, 7 cm (GE-Healthcare). The focused gel strips were subjected to second dimensional separation using NuPAGE Bis-Tris 4-12% Zoom gels (Invitrogen, Grand Island, NY), and subjected to colloidal Coomassie blue staining. Serum samples obtained from ^14^C-3-BrPA infused rats were resolved on SDS-PAGE and 2D gels, and subsequently incubated with “Amplify” solution (GE-Healthcare) prior to vacuum drying and exposed to X-ray film (GE-Healthcare) to obtain the images of autoradiogram. Only the authorized personnel handled all procedures involving ^14^C-3-BrPA, and appropriate radioactive decontaminations/containments were followed strictly according to the Johns Hopkins Radiation Safety Rules and Regulations.

### Protein identification by liquid chromatography-tandem mass spectrometry (LC-MS/MS)

Protein spots from 2D-gels were proteolyzed with trypsin as described previously [21]. Digested peptides were extracted and subjected to vacuum drying in a Speedvac, followed by reconstitution in 5 μL of 2% acetonitrile/0.1% formic acid, for further analysis by liquid chromatography/tandem mass spectrometry (LC-MS/MS) using LTQ Orbitrap Velos (2) MS (Thermo Fisher Scientific, http://www.thermofisher.com).

Peptides were loaded on a 75 μm × 2.5 cm C18 (YMC*GEL ODS-A 12 nm S-10 μm) trap at 600 nL/min 0.1% formic acid (solvent A) and fractionated at 300 nL/min on a 75 μm × 100 mm Magic C18 AQ 5 μm reverse-phase column (5 μm, 120 Å, Microm Bioresources, http://www.michrom.com) using a 3-10% solvent B (90% acetonitrile in 0.1% formic acid) gradient over 40 min. Eluting peptides were sprayed into an LTQ Orbitrap Velos mass spectrometer (ThermoScientific, http://www.thermo.com/orbitrap) through 1 μm emitter tip (New Objective, http://www.newobjective.com) at 2.0 kV. Survey scans (full ms) were acquired within 350–1700 m/z with up to 10 peptide masses (precursor ions) individually isolated at IW1.9 Da, and fragmented (MS/MS) using HCD 35 activation collision energy. Precursor and the fragment ions were analyzed at resolution 30,000 and 15,000, respectively. Dynamic exclusion of 30 s, repeat count 1, MIPS (monoisotopic ion precursor selection) “on”, m/z option “off”, lock mass “on” (silocsane 371 Da) were used.

For data analysis, tandem mass spectra were extracted, charge state deconvoluted and deisotoped by Proteome Discoverer (v1.3 Thermo Fisher Scientific). All MS/MS spectra were analyzed with Mascot v.2.2 Matrix Science, London, UK (http://www.matrixscience.com) using the NCBI 167nr Database, *Rattus* species with acquired raw MS/MS data, trypsin as enzyme, missed cleavage 1, precursor mass tolerance 10 ppm, fragment mass tolerance 0.02 Da, y, b ions, and oxidation on methionine as variable modifications. For each sample, Mascot search result *.dat files for nodes with/without extract were processed in Scaffold (http://www.proteomesoftware.com) combined as MUDPIT experiment to validate protein and peptide identifications.

## Abbreviations

3-BrPA: 3-bromopyruvate; LC-MS/MS: Liquid chromatography-tandem mass spectrometry; α1-AT: Alpha1 antitrypsin; 2D: gel electrophoresis: Two-dimensional gel electrophoresis; TUNEL: Terminal deoxynucleotidyl transferase dUTP nick-end labeling.

## Competing interests

Dr. Geschwind is the founder of Presciencelabs LLC, a biotech firm currently developing 3-BrPA for clinical use in liver cancer.

## Authors’ contributions

RK carried out the experiments such as immunohistochemical staining, 2D gel electrophoresis, spectroscopy analysis and drafted the manuscript. JG performed the conception and participated in the experimental design, and edited the manuscript. PR performed *in vivo* studies. TB and RC carried out the mass spectrometry analysis of 2D gel spots and data interpretation. SG-K conceived and designed the experiments, performed tissue and gel autoradiography, and drafted the manuscript. All authors read and approved the final manuscript.

## Supplementary Material

Additional file 1: Figure S1 A schematic showing the surgical procedure for *in vivo* delivery of 3-BrPA and blood draw in Sprague Dawley rat. **Figure S2**. Spectral analysis of mouse serum with and without 3-BrPA *in vivo*. (A). Absorption maxima of 3-BrPA dissolved in saline, at low concentration (0.06 mM), near IC_50_ concentration (0.2 mM) and *in vivo* therapeutic dose (1.75 mM). (B) Spectrum showing an increase in the peak intensity at 412 nm in the serum of mouse dosed with 3-BrPA (60 and 120 μM). (C) Spectrum showing an increase in the peak intensity at 538 and 572 nm in the serum of mouse dosed with 3-BrPA (60 and 120 μM). Click here for file
