# Supplementary material for: Systemic administration of 3-bromopyruvate reveals its interaction with serum proteins in a rat model
Source: BMC Res Notes. 2013 Jul 17;6:277. doi: 10.1186/1756-0500-6-277 (PMC3728150; doi:10.1186/1756-0500-6-277)

**Additional file 1**

**Figure S1.** A schematic showing the surgical procedure for *in vivo* delivery of 3-BrPA and blood draw in Sprague Dawley rat.

**
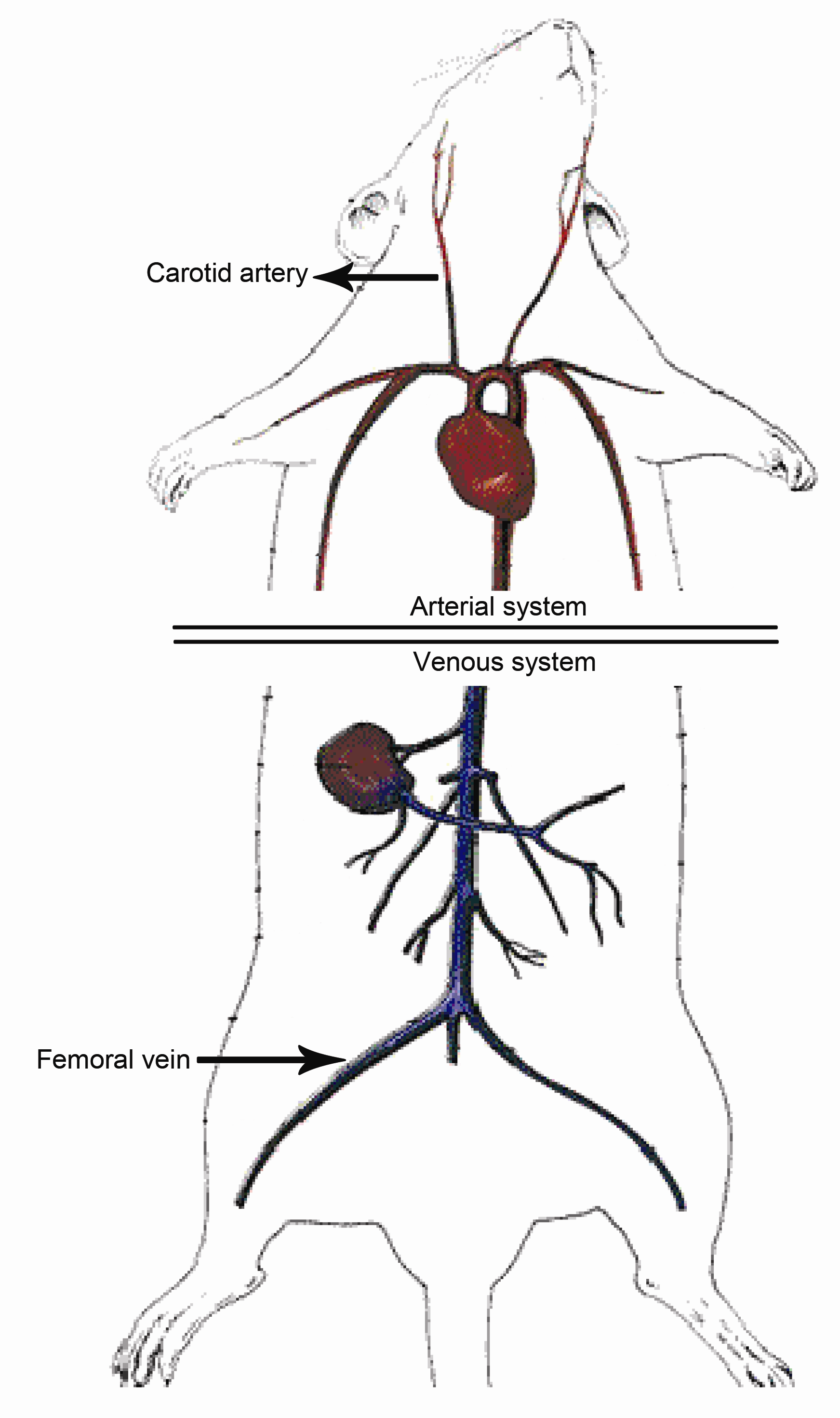
**

**Figure 2. Spectral analysis of mouse serum with and without 3-BrPA *in vivo*.** (A). Absorption maxima of 3-BrPA dissolved in saline, at low concentration (0.06mM), near IC_50_ concentration (0.2 mM) and *in vivo* therapeutic dose (1.75 mM). (B) Spectrum showing an increase in the peak intensity at 412 nm in the serum of mouse dosed with 3-BrPA (60 and 120 μM). (C) Spectrum showing an increase in the peak intensity at 538 and 572 nm in the serum of mouse dosed with 3-BrPA (60 and 120 μM).


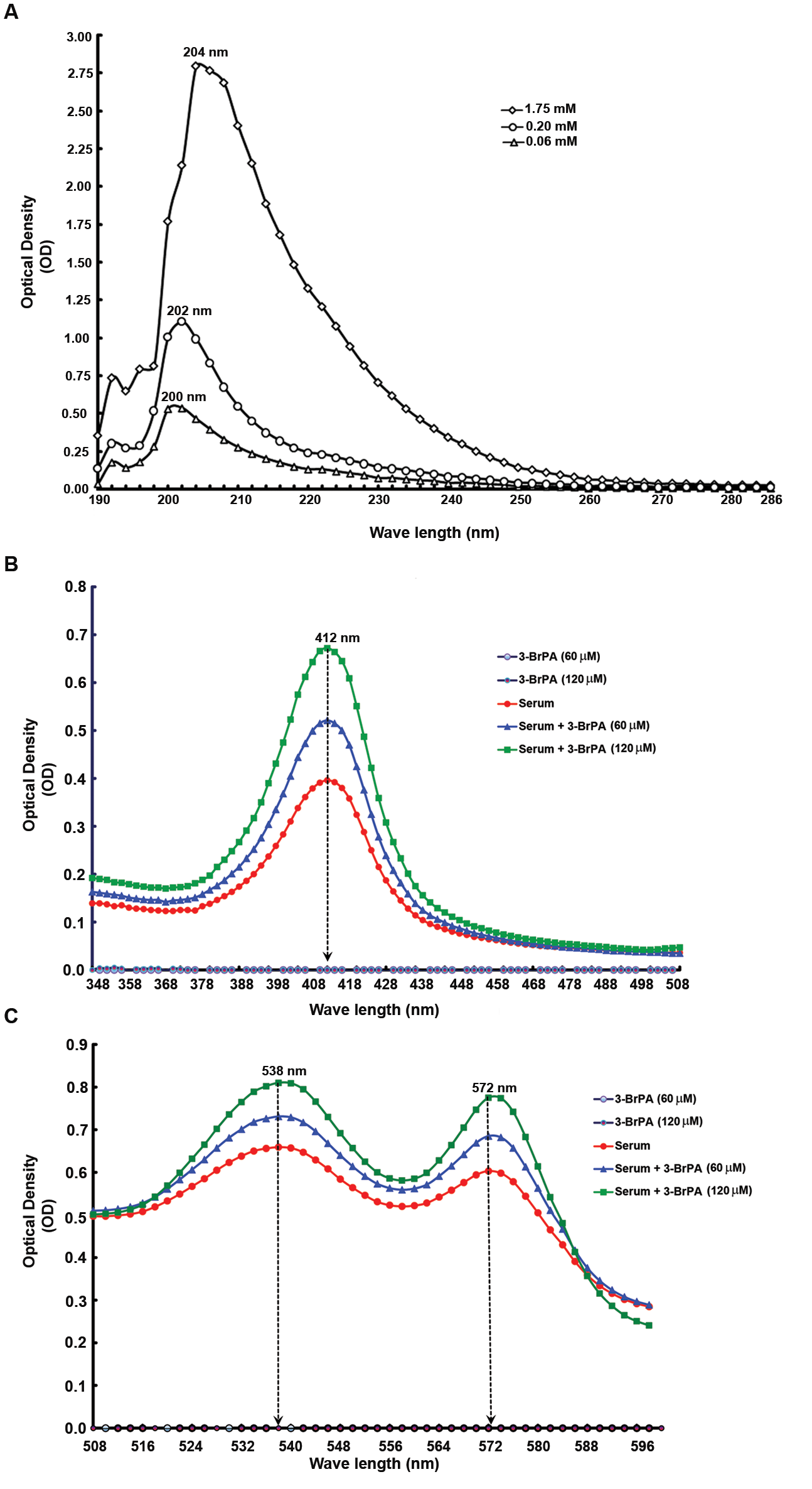

Supplement: Additional file 1: Figure S1 — A schematic showing the surgical procedure for in vivo delivery of 3-BrPA and blood draw in Sprague Dawley rat. Figure S2. Spectral analysis of mouse serum with and without 3-BrPA in vivo. (A). Absorption maxima of 3-BrPA dissolved in saline, at low concentration (0.06 mM), near IC50 concentration (0.2 mM) and in vivo therapeutic dose (1.75 mM). (B) Spectrum showing an increase in the peak intensity at 412 nm in the serum of mouse dosed with 3-BrPA (60 and 120 μM). (C) Spectrum showing an increase in the peak intensity at 538 and 572 nm in the serum of mouse dosed with 3-BrPA (60 and 120 μM). [file 1756-0500-6-277-S1.doc]
